# Supplementary material for: Dynamic optimization reveals alveolar epithelial cells as key mediators of host defense in invasive aspergillosis
Source: PLoS Comput Biol. 2021 Dec 13;17(12):e1009645. doi: 10.1371/journal.pcbi.1009645 (PMC8699926; doi:10.1371/journal.pcbi.1009645)
Supplement: S1 Appendix — Documentation of parameter estimation and calculation. (PDF) [file pcbi.1009645.s001.pdf]

S1 Appendix

Documentation of parameter estimation and calculation

$L(0), L_0$ Average number of AEC (type I and II) per alveolus. The total number of AEC in several mammalian species was elucidated by Stone et al. [1] and reports for murine lungs $11.6 + 14.8 = 26.4 \cdot 10^6\, cells$ (type I and II). Knust et al. [2] determined $2.31 \cdot 10^6$ alveoli for mice with a comparable weight ($20\, g$) and we estimate $L(0) = L_0 = \frac{26.4}{2.31} = 11.43\, cells.$

l_1 Rate of hyphae destroying AEC. In [3], epithelial damage was assessed in mice by LDH release in bronchial lavage showing a 2.5 fold change of damage after 24h. The rate is calculated by $l_1 = \frac{ln(fold\, change)}{\cdot 24h} = 0.0382\, cells^{-1}\, h^{-1}$.

l_2 Rate of neutrophils damaging AEC. In [4], neutrophils were stimulated *in vivo* with LPS and cell damage in lungs was assessed as free elastase activity in BALF. The relative damage was 72% after 12h and we estimate $l_2 = \frac{ln(1-0.72)}{12h} = 0.1061\, cells^{-1}\, h^{-1}$

l_3 As l_2 but for AM. Host damage mediated by AM is less frequently measured and we therefore rely on data of Hirano [5]. In that study rat epithelial cells (SV40T2) were co-cultured with rat alveolar macrophages and stimulated by LPS. The damage was determined by transepithelial resistance and dropped from $11\, \Omega cm^2$ to $0.5\, \Omega cm^2$ within 24h. To this end, we calculate $l_3 = \dfrac{ln(\frac{11}{0.5})}{24h} = 0.1288\, cells^{-1}\, h^{-1}$.

$R(0), R_0$ Initial dose of resting conidia. Value depends on the scenarios. *In vivo* experiments with mice typically challenged with 10^5-10^7 conidia. Therefore, alveolus occupation is typically 0.1 - 10 conidia per alveolus. A detailed calculation is also provided by Blickensdorf et al. [6].

r_1 Average time for swelling of conidia. After 3h the conidial rodlet layer is disrupted [7].

s_1 Average time for germination of swollen conidia. Total time from resting conidia to germination is on average 7h [8]. Therefore $s_1 = 7h - r_1 = 4h$.

s_2 Killing rate of swollen conidia by AM. Intensively studied, but rates show high variation across studies [9]. In contrast, the killing rate of conidia by AEC is more rarely calculated. However, in [10] both was investigated. Accordingly, murine AM showed faster killing than AEC along all timepoints. For murine AM, conidia survival was 0.6% after 12h of interaction and we calculate $s_2 = \frac{ln(0.006)}{12h} = 0.4263\, cells^{-1}\, h^{-1}$.

s_3 Killing rate of swollen conidia by neutrophils. Phagocytosis and killing rate of conidia by neutrophils have been frequently studied. However, in the study of Alflen et al. [11] the killing rate of conidia and hyphae was compared and murine neutrophils were used. Conidial viability dropped to 39% after 4h of co-incubation and we calculate $s_3 = -\frac{0.39}{4h} = 0.2354\, cells^{-1}\, h^{-1}$.

s_4 Killing rate of swollen conidia by AEC. From the same study as for s_2 [10], AEC possessed a slower killing rate and we calculate $s_2 = \frac{ln(0.065)}{12h} = 0.2278\, cells^{-1}\, h^{-1}$.

h_1 Growth rate of hyphae. The increase of hyphal length with time is a good estimator for doubling times of cells since cell size is rather constant. In glucose medium and at $37°$C the length of hyphae doubles every 1h [12]. The rate is then $h_1 = \frac{ln(2}{1h} = 0.6533\, h^{-1}$.

h_2 Killing rate of hyphae by neutrophils. From the same study as used to estimate s_3 [11], the viability of hyphae was measured after co-incubation with neutrophils. After 4h 68% of initial fungal cells were viable, which equals a rate of $-\frac{0.68}{4h} = 0.0964\, cells^{-1}\, h^{-1}$. However, hyphae are growing at the same time and therefore we estimate $h_2 = h_1 \cdot 1\, cells + 0.0964$ since neutrophil killing outpaces hyphal growth by the above calculated rate during a fungal challenge of one fungal cell per alveolus.

$M(0)$ Number of resident AM per alveolus in an uninfected host. Recently, Amich et al. [13] were able to use 3D light sheet fluorescence microscopy to investigate invasive aspergillosis in *in vivo* mouse models. Due to this imaging approach one can precisely determine the number of immune cells within an alveolus. On average they find $M(0) = 0.2926\, cells$ AM per alveolus. Note, that in the agent-based model of Blickensdorf et al. [6] a slightly higher number of AM per murine alveolus (0.74) was calculated based on whole tissue cell counts [1].

m_1 Rate of recruitment of AM by influx and maturation of monocytes or even proliferation of resident macrophages. To complement data used for $M(0)$ and $N(0)$, we estimate the recruitment rates of AM and neutrophils based on data from a previous publication [14] of the same group of authors, which performed 3D light sheet fluorescence microscopy [13]. In that publication a similar murine infection model of invasive aspergillosis was used, but cell numbers were quantified by fluorescence-activated cell sorting (FACS) of lung tissue samples. For AM a rise from 2.75 to $9\, 10^5 cells$ in 4h after a mild dose of conidia was reported. We estimate $m_1 = M(0)\frac{9}{2.75 \cdot 4h} = 0.2394\, cells\, h^{-1}$.

m_2 Rate of alveolar macrophage depletion. Based on the same study as m_1 [14] macrophage level was on control level after 12h of peak inflammation. So we calculate $m_2 = M(0)\frac{9}{2.75 \cdot 12h} = 0.0798\, cells\, h^{-1}$.

m_3 Lysis rate of AM by swollen conidia. While phagocytosis and killing rate by AM are frequently reported the *vice versa* scenario is rarely depicted. For mice cytotoxicity of swollen conidia was reported [15] to be 84% after 24 h that corresponds to a rate of $m_3 = -\frac{ln(1-0.84)}{\cdot 24} = 0.0764\, cells^{-1}\, h^{-1}$.

c_1 Release rate of pro-inflammatory cytokines by AM. We intentionally do not model a single cytokine due to the complex network of cytokine functions and signaling to recruit neutrophils. Based on several studies investigating the time course of prominent pro-inflammatory cytokines we decided to set the cytokine release rate to $c_1 = 1\, cells^{-2}\, h^{-1}$ to mimic the peak in cytokine concentration at 5-10h after infection or stimulation with LPS.

c_2 Release rate of pro-inflammatory cytokines by lung AEC. Cytokine release of lung AEC is not well understood and only covered by few studies. A pioneering study [16] investigated the potential of human lung AM and AEC to recruit neutrophils when stimulated by LPS. Since AEC showed a 43.75% higher number of recruited neutrophils under similar conditions, we set $c_2 = 1.14375\, cells^{-2}\, h^{-1}$.

c_3 Decay rate of pro-inflammatory cytokines. As for c_1 we estimated the decay rate based on the investigation of several time courses and observed that decay is slower than release. For reference we set $c_3 = 0.1\, h^{-1}$.

$N(0)$ Number of tissue resident neutrophils. As described for $M(0)$ microscopy data of murine lungs reported a neutrophil density of $N(0) = 0.2628\, cells$ neutrophils per alveolus [13].

n_1 Rate of neutrophil recruitment by influx of leukocytes. Similar to the definition of m_1 an increase of neutrophils is reported in [14] from $1.9 \cdot 10^5 cells$ to $7.6 \cdot 10^5 cells$. To this end, we calculate $n_1 = N(0)\frac{7.6\cdot}{1.9\cdot 4h} = 0.2628\, cells\, h^{-1}$.

n_2 Rate of neutrophil depletion. Based on the same study as n_1 [14] neutrophil level was on control level after 12h of peak inflammation. So we calculate $n_2 = N(0)\frac{7.6\cdot}{2.8\cdot 12h} = 0.0594 cells\, h^{-1}$.

n_3 Lysis rate of neutrophils by fungal cells. In [17] induced decondensation of nuclei of neutrophils ($1 \cdot 10^6 cells$) was measured in response to short hyphae ($750 CFU$ with length of $10 - 100 \mu m$). After 6h of co-incubation 28 % of neutrophil nuclei were decondensed. Based on the assumption that hyphae contain around 10 nuclei at the reported length, we estimate an MOI of 1:10 and calculate the rate as $n_3 = -\frac{ln(1-0.28)}{0.1\cdot 6h} = 0.5475\, cells^{-1}\, h^{-1}$.

References

[1] K. C. Stone, R. R. Mercer, P. Gehr, B. Stockstill, J. D. Crapo, et al., Allometric relationships of cell numbers and size in the mammalian lung, American Journal of Respiratory Cell and Molecular Biology 6 (2) (1992) 235–43.

[2] J. Knust, M. Ochs, H. J. G. Gundersen, J. R. Nyengaard, Stereological estimates of alveolar number and size and capillary length and surface area in mice lungs, The Anatomical Record: Advances in Integrative Anatomy and Evolutionary Biology: Advances in Integrative Anatomy and Evolutionary Biology 292 (1) (2009) 113–122.

[3] M. Bertuzzi, M. Schrettl, L. Alcazar-Fuoli, T. C. Cairns, A. Muñoz, L. A. Walker, S. Herbst, M. Safari, A. M. Cheverton, D. Chen, et al., The pH-responsive PacC transcription factor of *Aspergillus fumigatus* governs epithelial entry and tissue invasion during pulmonary aspergillosis, PLoS Pathogens 10 (10) (2014) e1004413.

[4] M. Saffarzadeh, C. Juenemann, M. A. Queisser, G. Lochnit, G. Barreto, S. P. Galuska, J. Lohmeyer, K. T. Preissner, Neutrophil extracellular traps directly induce epithelial and endothelial cell death: a predominant role of histones, PloS One 7 (2) (2012) e32366.

[5] S. Hirano, Interaction of rat alveolar macrophages with pulmonary epithelial cells following exposure to lipopolysaccharide, Archives of Toxicology 70 (3-4) (1996) 230–236.

[6] M. Blickensdorf, S. Timme, M. T. Figge, Comparative Assessment of Aspergillosis by Virtual Infection Modeling in Murine and Human Lung, Frontiers in Immunology 10.

[7] E. Dague, D. Alsteens, J.-P. Latgé, Y. F. Dufrêne, High-resolution cell surface dynamics of germinating *Aspergillus fumigatus* conidia, Biophysical journal 94 (2) (2008) 656–660.

[8] K. J. Kwon-Chung, J. A. Sugui, *Aspergillus fumigatus*—what makes the species a ubiquitous human fungal pathogen?, PLoS pathogens 9 (12) (2013) e1003743.

[9] B. Philippe, O. Ibrahim-Granet, M. Prevost, M. Gougerot-Pocidalo, M. S. Perez, A. Van der Meeren, J. Latge, Killing of *Aspergillus fumigatus* by alveolar macrophages is mediated by reactive oxidant intermediates, Infection and Immunity 71 (6) (2003) 3034–3042.

[10] J. A. Wasylnka, M. M. Moore, *Aspergillus fumigatus* conidia survive and germinate in acidic organelles of A549 epithelial cells, Journal of Cell Science 116 (8) (2003) 1579–1587.

[11] A. Alflen, S. Prüfer, K. Ebner, S. Reuter, P. A. Lopez, I. Scharrer, F. Banno, M. Stassen, H. Schild, K. Jurk, et al., ADAMTS-13 regulates neutrophil recruitment in a mouse model of invasive pulmonary aspergillosis, Scientific Reports 7 (1) (2017) 7184.

[12] T. T. Ng, G. D. Robson, D. W. Denning, Hydrocortisone-enhanced growth of *Aspergillus spp.*: implications for pathogenesis, Microbiology 140 (9) (1994) 2475–2479.

[13] J. Amich, Z. Mokhtari, M. Strobel, E. Vialetto, D. Sheta, Y. Yu, J. Hartweg, N. Kalleda, K. J. Jarick, C. Brede, et al., Three-dimensional light sheet fluorescence microscopy of lungs to dissect local host immune-*Aspergillus fumigatus* interactions, mBio 11 (1).

[14] N. Kalleda, J. Amich, B. Arslan, S. Poreddy, K. Mattenheimer, Z. Mokhtari, H. Einsele, M. Brock, K. G. Heinze, A. Beilhack, Dynamic immune cell recruitment after murine pulmonary *Aspergillus fumigatus* infection under different immunosuppressive regimens, Frontiers in Microbiology 7 (2016) 1107.

[15] S. Slesiona, M. Gressler, M. Mihlan, C. Zaehle, M. Schaller, D. Barz, B. Hube, I. D. Jacobsen, M. Brock, Persistence versus escape: *Aspergillus terreus* and *Aspergillus fumigatus* employ different strategies during interactions with macrophages, PloS One 7 (2) (2012) e31223.

[16] A. J. Thorley, P. A. Ford, M. A. Giembycz, P. Goldstraw, A. Young, T. D. Tetley, Differential regulation of cytokine release and leukocyte migration by lipopolysaccharide-stimulated primary human lung alveolar type II epithelial cells and macrophages, The Journal of Immunology 178 (1) (2007) 463–473.

[17] M. Röhm, M. J. Grimm, A. C. D'Auria, N. G. Almyroudis, B. H. Segal, C. F. Urban, NADPH oxidase promotes neutrophil extracellular trap formation in pulmonary aspergillosis, Infection and Immunity 82 (5) (2014) 1766–1777.
