# Supplementary material for: Dynamic optimization reveals alveolar epithelial cells as key mediators of host defense in invasive aspergillosis
Source: PLoS Comput Biol. 2021 Dec 13;17(12):e1009645. doi: 10.1371/journal.pcbi.1009645 (PMC8699926; doi:10.1371/journal.pcbi.1009645)
Supplement: S2 Appendix — Influence of weighting innate immune response objectives. (PDF) [file pcbi.1009645.s002.pdf]

S2 Appendix

Influence of weightings in the innate immune response objectives.

Model behavior with no fungal burden minimization and only minimization of tissue damage.

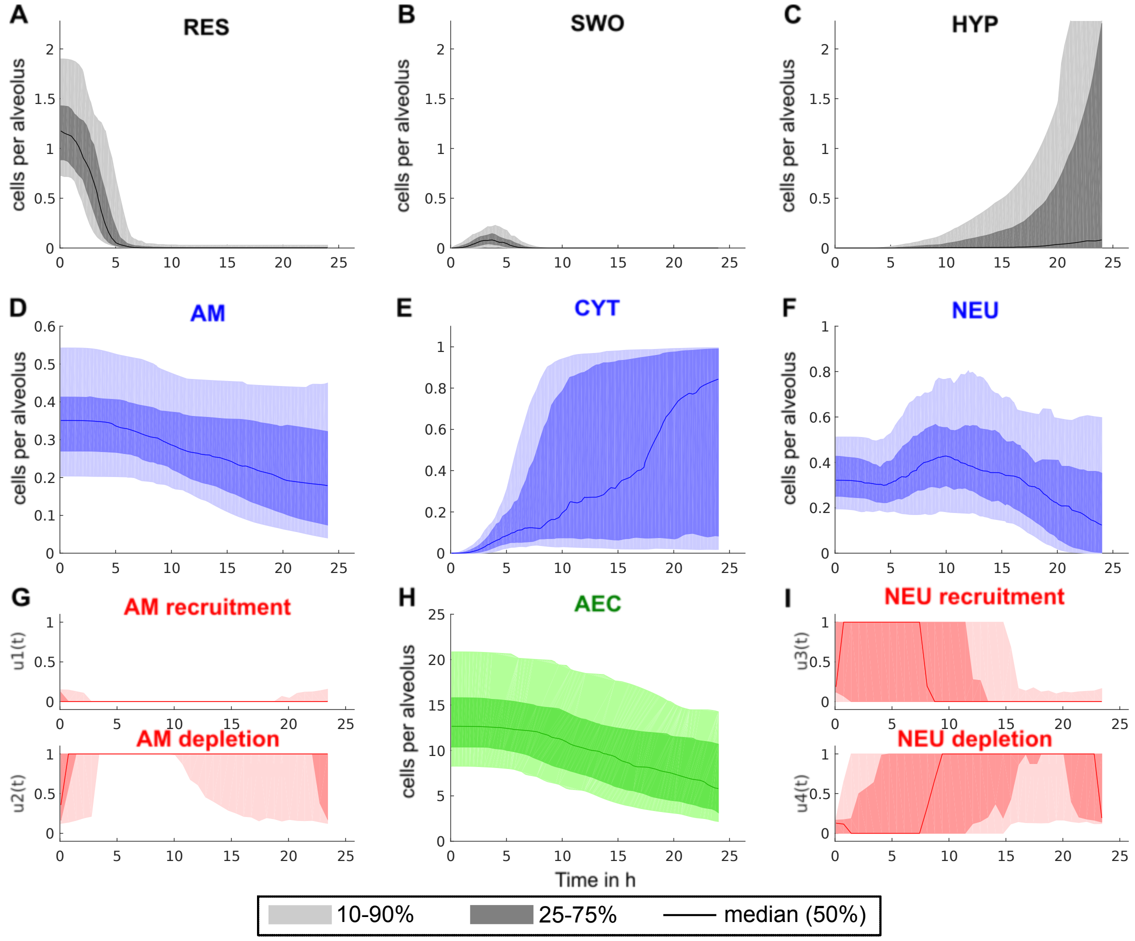

Figure A: Dynamics of innate immune response of the murine host challenged with a high dose of conidia (one per alveolus). The solution space of 100 parameter sets is depicted with shadings indicating the confidence intervals of time courses. Colors as in previous figures and equations.

Model behavior with only fungal burden minimization and no minimization of tissue damage.

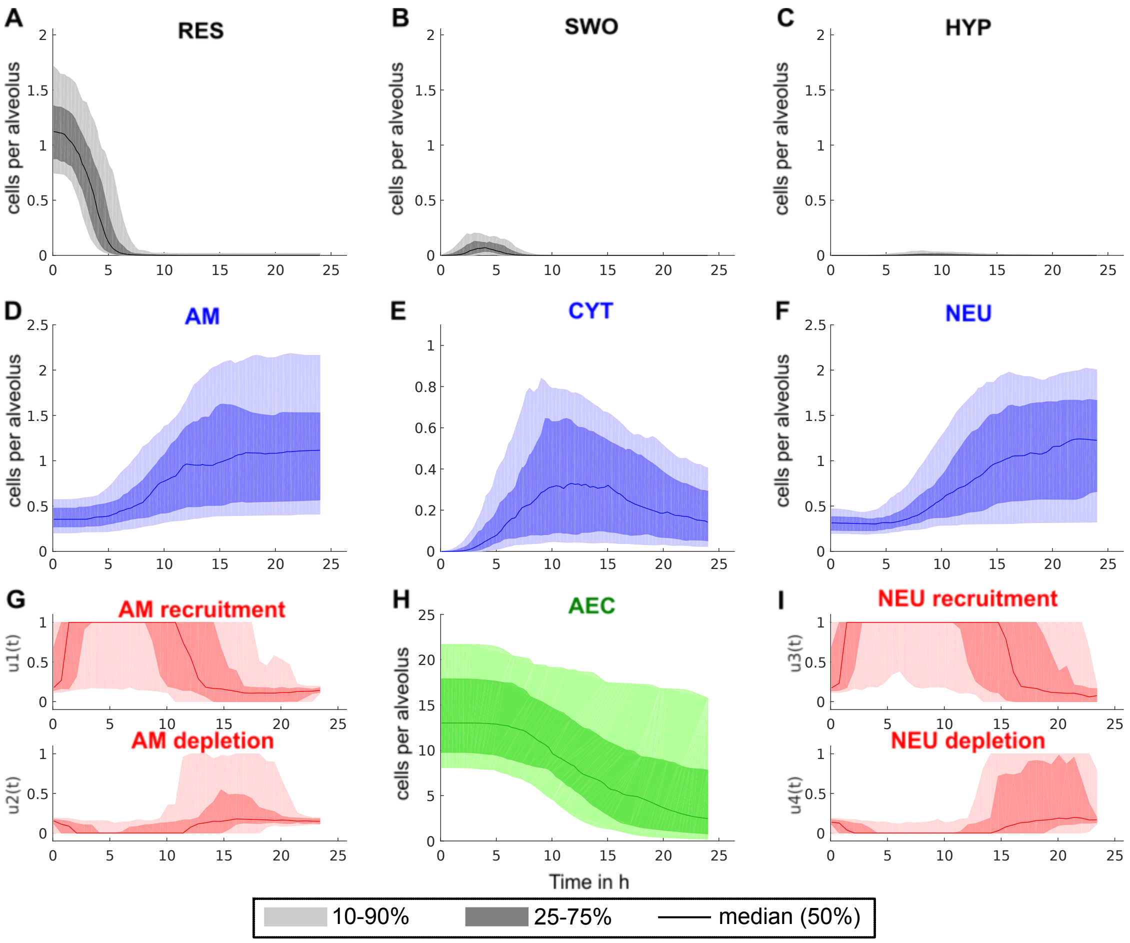

Figure B: Dynamics of innate immune response of the murine host challenged with a high dose of conidia (one per alveolus). The solution space of 100 parameter sets is depicted with shadings indicating the confidence intervals of time courses. Colors as in previous figures and equations.

Parameter sensitivity and different objective function weightings

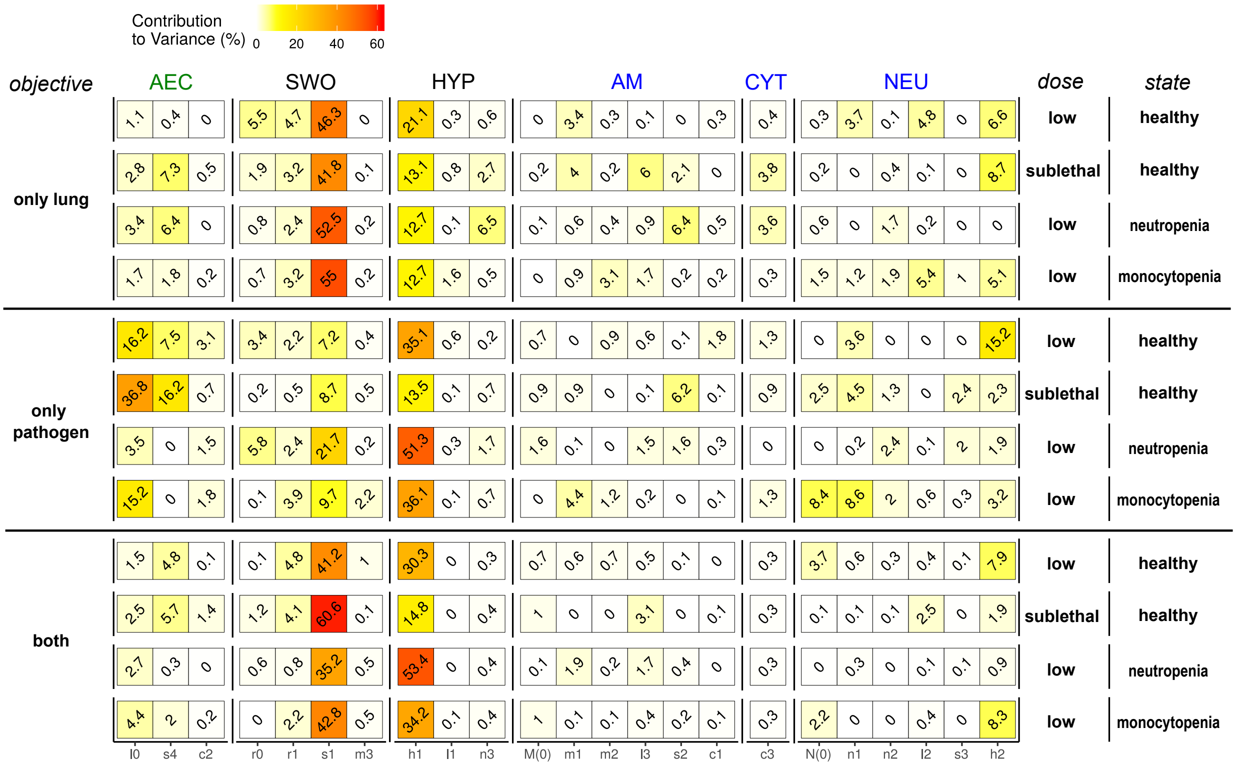

Figure C: Influence of parameters on the severeness of infection depicted by the contribution to variance (brown). This relative contribution is based on Spearman rank correlation of parameter value and objective value of the optimal solution.
