# Supplementary figures and images for: Dynamic optimization reveals alveolar epithelial cells as key mediators of host defense in invasive aspergillosis
Source: PLoS Comput Biol. 2021 Dec 13;17(12):e1009645. doi: 10.1371/journal.pcbi.1009645 (PMC8699926; doi:10.1371/journal.pcbi.1009645)

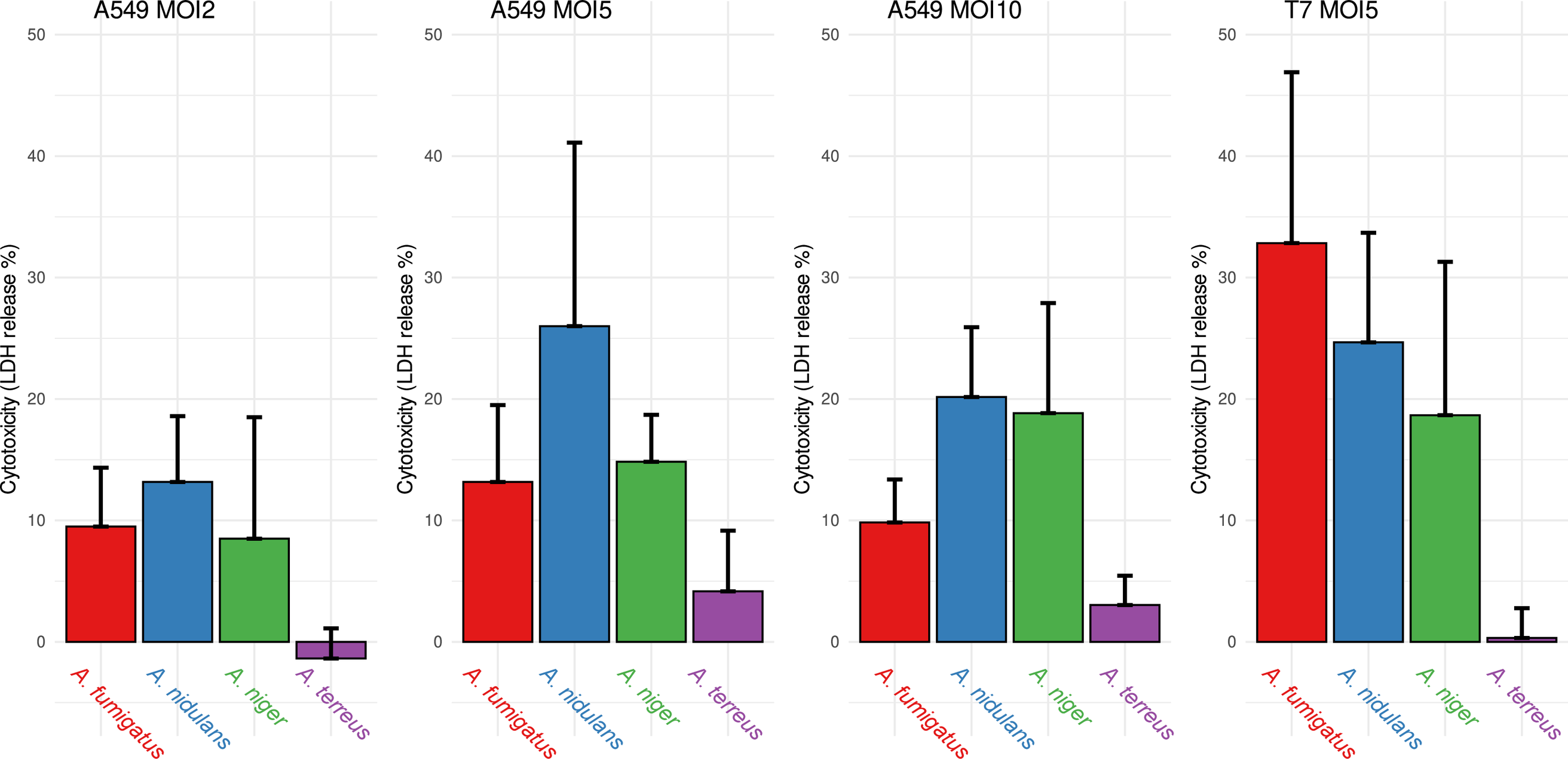

Supplement: S1 Fig — In addition to the depicted LDH release measurements of epithelial cells upon 24h co-incubation with Aspergillus spp. at an MOI = 5 (main text Fig 4D and 4E), cytotoxicity was measured for MOI = 2 and MOI = 10 for human A549 cells to demonstrate the influence of fungal burden. (TIF) [file pcbi.1009645.s004.tif]
